# Screening for oropharyngeal dysphagia in hospitalized COVID-19 patients: a prospective study

**DOI:** 10.1007/s00405-022-07810-z

**Published:** 2023-01-06

**Authors:** Ahmed Mohamed Zayed, Omayma Afsah, Tamer Elhadidy, Tamer Abou-Elsaad

**Affiliations:** 1 Phoniatric Unit, ORL Department, Faculty of Medicine, Mansoura University, Mansoura, 35516 Egypt; 2 Chest Diseases Department, Faculty of Medicine, Mansoura University, Mansoura, Egypt

**Keywords:** COVID-19, Oropharyngeal, Dysphagia, Screening, Swallowing

## Abstract

**Purpose:**

To screen for oropharyngeal dysphagia (OD) in hospitalized COVID-19 patients.

**Methods:**

A descriptive longitudinal study was conducted on 500 adult patients with confirmed COVID-19 in the age range of 19–65 years who were admitted to the main university isolation hospital (whether admitted in the ward or the intensive care unit). Screening for OD was done using the Arabic version of the Eating Assessment Tool (EAT-10) and the Yale swallow protocol.

**Results:**

45.4% of the admitted and 40.97% of the discharged COVID-19 patients had a positive screen for OD. Several risk factors for OD could be detected. These include older age, longer duration of presenting symptoms of COVID-19, presence of ageusia and anosmia, presence of dysphonia, ICU admission, lower oxygen saturation, higher respiratory rate, presence of OD at admission, longer duration of hospital stay, and use of noninvasive ventilation (NIV) and/or invasive mechanical ventilation (IMV).

**Conclusions:**

Screening for OD in hospitalized COVID-19 patients is a mandatory procedure, whether for admitted or discharged patients.

## Introduction

Coronavirus disease 2019 (COVID-19) was initially discovered in Wuhan City, China, in December 2019 [1]. COVID-19 illness is caused by severe acute respiratory syndrome coronavirus 2 (SARS-CoV-2) [2]. According to reviews and meta-analyses, the key clinical manifestations of COVID-19 were fever, dry cough, and apparent lung abnormalities on chest computed tomography images. In addition, some patients may encounter loss of taste and smell, fatigue, and muscle ache. Severe cases may develop acute respiratory distress syndrome, multi-organ failure [3–6], and/or neurological sequelae, such as Guillain–Barré syndrome or stroke [7, 8].

Dysphagia is the difficulty or inability to swallow resulting from any abnormality affecting the structure or function of normal deglutition. Lechien et al. [9] stated that “the pathophysiology of oropharyngeal dysphagia (OD) in COVID-19 patients is assumed to be related to the interaction of the virus with angiotensin-converting enzyme 2 (ACE2) and transmembrane protease serine 2 (TMPRSS2), proteins that are present in relevant anatomic regions for swallowing function, such as oral, pharyngeal and nasal human mucosa”. One of the main pathophysiological factors for SARS-CoV-2-related oropharyngeal dysphagia is oropharyngeal sensory impairment, likely linked to glossopharyngeal and vagal sensory neuropathy [10].

Swallowing and breathing are closely interconnected and highly coordinated functions [11]. Patients with COVID-19 have varying degrees of dyspnea, ranging from 18.6% to 59% [12]. This dyspnea might compromise airway protection resulting from the disturbance to tight synchronization of swallowing and respiration [13]. COVID-19 is associated with a high incidence of critical illness, a risk factor for swallowing difficulties [14]. The intensive care unit (ICU)-acquired weakness (caused by disuse, sedation, or neuromuscular blocking agents) may affect the swallowing muscles [15]. In 94% of cases, forms of invasive mechanical ventilation, such as prolonged orotracheal intubation, can cause laryngeal injury and/or dysphagia [16, 17]. Heavy sedation for prolonged intubation heightens the risk of pharyngeal dysfunction and uncoordinated swallowing and breathing [18].

There is emerging evidence about the association between COVID-19 and dysphagia. The primary aim of this study was to screen for oropharyngeal dysphagia in patients infected with COVID-19 who were admitted to an isolation hospital. The secondary purpose was to identify the possible risk factors of oropharyngeal dysphagia in hospitalized COVID-19 patients.

## Subjects and methods

### Subjects

A descriptive longitudinal study was conducted on 500 adult patients with confirmed COVID-19 [by detection of SARS-CoV-2 RNA by reverse transcription–polymerase chain reaction (RT–PCR)] who were admitted to the main university isolation hospital (whether admitted in the ward or the ICU) during the period from May 2021 to December 2021. The main reasons for admission were low oxygen saturation (< 94%) on room air and dyspnea.

### Exclusion criteria

- Patients aged < 18 years and > 65 years.
- Patients on invasive mechanical ventilation at the time of hospital admission.
- Patients with impaired level of consciousness (Glasgow coma score less than 13).
- Stroke patients with oropharyngeal dysphagia (whether recent stroke during COVID-19 infection or old stroke).
- Patients with a history of oropharyngeal dysphagia due to other medical conditions, such as head and neck cancer or other neurological disorders.
- Tracheostomized patients.

### Methods

All individuals participating in this study were subjected to the following protocol of assessment:

(A) History taking:

- Demographic data (name, chronological age, and gender).

- Medical history: including a history of stroke or other neurological disorders, head and neck cancer, or previous history of oropharyngeal dysphagia.

- Present history: including.

* Duration of the presenting symptoms of COVID-19.

* Presence or absence of ageusia and anosmia (at the initial time of hospital admission and at the time of hospital discharge).

* Presence or absence of dysphonia (at the initial time of hospital admission and at the time of hospital discharge).

* Current oxygen therapy (Conventional oxygen therapy/non-invasive ventilation).

(B) Clinical examination: including measurement of oxygen saturation by pulse oximetry, respiratory rate, and heart rate to assess the respiratory status of the patients.

(C) Assessment of orofacial muscles and cranial nerves was done by observing the muscles at rest and during speech.

(D) Screening for OD: this was done twice at the initial time of hospital admission and at the time of hospital discharge. Two screening tools were used:

(1) Patient Questionnaire—The Arabic version of the Eating Assessment Tool (EAT-10) [19]:

**-** The Arabic version of the Eating Assessment Tool (EAT-10) is a self-administered screening tool for the subjective assessment of oropharyngeal dysphagia in the Arabic-speaking population. It consists of 10 statements, each is scored on a 5-point rating scale as follows: 0 = No problem, 1 = Mild problem, 2 = Mild to moderate, 3 = Moderate problem, and 4 = Severe problem.

- A summated EAT-10 total score ranges from 0 to 40, with a score ≥ 3 indicative of dysphagia. An elevated EAT-10 score points to a higher self-perception of dysphagia.

(2) Bedside (Non-instrumental) swallowing assessment: using the evidence-based Yale swallow protocol [20], which consists of 3 items:

I. Brief cognitive screen: what is your name? Where are you right now? What year is it?

II. Oral Mechanism Examination (Labial closure, Lingual range of motion, Facial symmetry (smile/pucker).

III. 3-oz water swallow test, on which the patient was judged to pass or fail.

according to the following criteria:

*PASS* Complete and uninterrupted drinking of 90 ml (3 oz) of water without overt signs of aspiration, i.e., coughing or choking, either during or immediately after completion.

*FAIL* Inability to drink the entire 3 oz in sequential swallows due to stopping/starting or the patient exhibited overt signs of aspiration, i.e., coughing or choking, either during or immediately after completion.

*Precautions* Owing to the increased probability of coughing in our patients during swallowing assessment, the use of appropriate personal protective equipment (PPE), such as an N95 mask, face shield/goggles, gown, and gloves, was considered mandatory as tiny respiratory droplets generated from the cough can remain suspended in air and maybe a potential source of transmission for the clinician [21]. The distance between the patient and the phoniatrician during the bedside swallowing assessment was about 2 m, and the phoniatrician was positioned to the side of the patient rather than face-to-face.

### Statistical analysis

Data were fed to the computer and analyzed using IBM SPSS software, version 22.0. Qualitative data were described using numbers and percentages. Quantitative data were described using median (minimum and maximum) and mean and standard deviation for parametric data after testing normality using the Kolmogorov–Smirnov test. The significance of the obtained results was judged at the (0.05) level. *p* value was considered statistically significant if < 0.05 and highly significant if < 0.01. The Chi-square test was used for the comparison of 2 or more groups. The student *t* test was used to compare two independent groups for parametric tests, and the Mann–Whitney *U* test was used to compare two independent groups for non-parametric tests. Binary stepwise logistic regression analysis was used to predict independent variables of a binary outcome. Significant predictors in the univariate analysis were entered into the regression model using the forward Wald method/Enter. Adjusted odds ratios and their 95% confidence interval were calculated.

## Results

### Descriptive statistics of the whole studied group

The present study was conducted on 500 patients, including 295 females (59%) and 205 males (41%) in the age range 19–65 years (mean 54.2 ± 11.03). 232 patients (46.4%) were admitted to the ward, while 268 patients (53.6%) were admitted to the intensive care unit (ICU). The mean duration of the presenting symptoms of COVID-19 before hospital admission was 9.04 ± 4.07 days. Dysphonia was evident in 43.4% of patients at the time of hospital admission. No significant abnormality was detected when assessing orofacial muscles and cranial nerves. Only 310 patients (62%) were discharged alive with a hospital stay of 4–42 days (median of 9 days). Out of the 500 admitted patients, 227 patients (45.4%) had a positive screen for oropharyngeal dysphagia (scored ≥ 3 on EAT-10 questionnaire and/or failed the 3-oz water swallow test). In contrast, out of the 310 discharged patients, 127 (40.97%) experienced OD (Tables 1 and 2).

**Table 1:** Demographic and basic data of the whole studied group (*N* = 500)

	Total number = 500
Age (years)	
Mean ± SD	54.22 ± 11.03
Duration of presenting symptoms (days)	
Median (range)	7 (3.0–21)
Oxygen saturation	
Mean ± SD	82.59 ± 7.18
Respiratory rate	
Mean ± SD	28.16 ± 3.84
Length of hospital stay for discharged alive patients (days) Median (range)	9.0 (4.0–42.0)

	Number (%)
Gender	
Male	205 (41%)
Female	295 (59%)
Aguesia and anosmia	151 (30.2%)
Dysphonia	217 (43.4%)
Place of admission	
ICU	268 (53.6%)
Ward	232 (46.4%)
Oropharyngeal dysphagia (OD)	
Absent	273 (54.6%)
Present	227 (45.4%)
Mortality	
Discharged alive	310 (62%)
Died	190 (38%)

**Table 2:** EAT-10 scores and Yale swallow protocol results of the whole studied group

	Number	Percentage
Admitted patients (*N* = 500)		
EAT-10 scores ≥ 3	184	36.8%
EAT-10 scores < 3	316	63.2%
Pass Yale swallow protocol	284	56.8%
Fail Yale swallow protocol (Aspiration risk)	216	43.2%
EAT-10 scores ≥ 3 and/or Fail Yale swallow protocol	227	45.4%
Discharged patients (*N* = 310)		
EAT-10 scores ≥ 3	127	40.97%
EAT-10 scores < 3	183	59.03%
Pass Yale swallow protocol	234	75.48%
Fail Yale swallow protocol (Aspiration risk)	76	24.52%
EAT-10 scores ≥ 3 and/or Fail Yale swallow protocol	127	40.97%

### Descriptive and comparative analysis of different assessment parameters in patients with OD and patients without OD

At the time of hospital admission, most patients with OD (84.6%) were admitted to the ICU, whereas most patients without OD (72.2%) were admitted to the ward. Patients with OD showed a significantly higher age range, longer duration of presenting symptoms, higher respiratory rate, lower mean oxygen saturation, a longer length of hospital stay, and higher mortality rate than patients without OD. The number of patients who presented with dysphonia, ageusia, and anosmia at the time of hospital admission and OD at the time of hospital discharge was significantly greater in the group of patients with OD than in the group of patients without OD (Table 3).

**Table 3:** Comparison of admitted patients with OD and without OD (*N* = 500)

	Patients without OD (*n* = 273)	Patients with OD (*n* = 227)	Test of significance
Age (years)			*t* = 4.19
Mean ± SD	52.36 ± 11.92	56.45 ± 9.39	*p* < 0.001*
Gender			
Male	121 (44.3%)	84 (37.0%)	*x*^2^ = 2.74
Female	152 (55.7%)	143 (63.0%)	*p* = 0.098
Duration of presenting symptoms (days)			*z* = 6.22
Median (range)	7(3–21)	10(3–21)	*p* < 0.001*
Aguesia and anosmia			
Absent	208 (76.2%)	141 (62.1%)	*x*^2^ = 1.65
Present	65 (23.8%)	86 (37.9%)	*p* = 0.001*
Dysphonia			
Absent	224 (82.1%)	59 (26%)	*x*^2^ = 158.57
Present	49 (17.9%)	168 (74%)	*p* = 0.001*
Oxygen saturation			*t* = 19.59
Mean ± SD	86.91 ± 4.06	77.41 ± 6.66	*p* < 0.001*
Respiratory rate			*t* = 28.04
Mean ± SD	25.42 ± 1.97	31.46 ± 2.83	*p* < 0.001*
Place of admission			
ICU	76 (27.8%)	192 (84.6%)	*x*^2^ = 160.46
Ward	197 (72.2%)	35 (15.4%)	*p* = 0.001*
Mortality			
Discharged alive	213 (78.0%)	97 (42.7%)	*x*^2^ = 65.52
Died	60 (22.0%)	130 (57.63%)	*p* < 0.001*
Length of hospital stay for discharged alive patients (days)			*z* = 3.56
Median (range)	8 (4–41)	11 (4–42)	*p* < 0.001*
Oropharyngeal dysphagia (OD) in discharged alive patients			
Absent	165 (77.5%)	18 (18.6%)	*x*^2^ = 95.63
Present	48 (22.5%)	79 (81.4%)	*p* < 0.001*

*t* Student *t* test, *x*^*2*^ Chi-square test, *z* Mann–Whitney *U* test

*p < 0.05: statistically significant

### Risk factors of oropharyngeal dysphagia in admitted patients

Statistically significant associations were found between OD in COVID-19 patients at the time of hospital admission and the following factors: older age, longer duration of presenting symptoms of COVID-19, presence of ageusia and anosmia, presence of dysphonia, ICU admission, lower oxygen saturation and higher respiratory rate. Patients with ageusia and anosmia had a risk of developing OD 0.5 times more than patients with intact taste and smell function, and dysphonic patients had a chance to develop OD 13 times more than non-dysphonic patients. Patients admitted to ICU had a risk of developing OD 14.22 times more than those admitted to the ward. Multivariate analysis was done for significant factors detected by univariate analysis and illustrated that the combined presence of dysphonia, presence of ageusia and anosmia, higher respiratory rate, and longer duration of presenting symptoms of COVID-19 were significant risk factors with an adjusted odds ratio (6.57, 2.35, 2.01 and 1.17, respectively) and the overall percentage predicted OD by those factors was 93% (Table 4). Patients with OD had a mortality risk of 4.75 times more than those without OD.

**Table 4:** Univariate and multivariate analyses of the risk factors of OD among the admitted patients (*N* = 500)

Variables	Total number	OD		Univariate analysis		Multivariate analysis	
				*p*	Crude Odds ratio (95% CI)	*p*	Adjusted odds ratio (95% CI)
		Absent (*N* = 273)	Present (*N* = 227)				
Age (years)							
Mean ± SD	500	52.36 ± 11.92	56.45 ± 9.39	< 0.001*	1.036 (1.02–1.06)	0.424	1.02(0.979–1.05)
Gender							
Male (R)	205	121 (59%)	84 (41%)	0.098	1.36 (0.945–1.942)		
Female	295	152 (51.5%)	143 (48.5%)				
Duration of presenting symptoms (days)							
Median (range)	500	7(3–21)	10(3–21)	< 0.001*	1.176 (1.12–1.24)	0.002*	1.167 (1.05–1.29)
Aguesia and anosmia							
Absent (R)	349	208 (59.6%)	141 (40.4%)	0.001*	0.512 (0.348–0.754)	0.036*	2.35 (1.06–5.21)
Present	151	65 (43%)	86 (57%)				
Dysphonia							
Absent (R)	283	224 (79.2%)	59 (20.8%)	0.001*	13.02 (8.48–19.98)	< 0.001*	6.57 (3.01–14.34)
Present	217	49 (22.6%)	168 (77.4%)				
Admission							
ICU	268	76 (28.4%)	192 (71.6%)	< 0.001*	14.22 (9.09–22.23)	0.124	1.98 (0.830–4.69)
Ward (R)	232	197 (84.9%)	35 (15.1%)				
Oxygen saturation	500	86.91 ± 4.06	77.41 ± 6.66	< 0.001*	0.735 (0.696–0.774)	0.057	0.921 (0.847–1.0)
Respiratory rate	500	25.42 ± 1.97	31.46 ± 2.83	< 0.001*	2.44 (2.09–2.84)	< 0.001*	2.01 (1.66–2.43)
	**Overall % predicted = 93%** **Model *χ***^**2**^ = 549.6, *p* < 0.001*						

*R* reference group

*p < 0.05: statistically significant and should be in bold

### Risk factors of oropharyngeal dysphagia in discharged patients

Statistically significant associations were found between OD in COVID-19 patients at the time of hospital discharge and the following factors: older age, presence of OD at admission, longer duration of hospital stay, presence of dysphonia, ICU admission, and use of noninvasive and/or invasive mechanical ventilation. Patients with OD at admission were risky for OD at the time of discharge 16.92 times more than patients without OD at admission; also, dysphonic patients had a risk of developing OD 15.13 times more than non-dysphonic patients. Invasive ventilation was a risk factor for OD more than non-invasive ventilation, since seven (100%) of patients weaned from invasive mechanical ventilation developed OD. On the other hand, when comparing non-invasive ventilation with conventional oxygen therapy, patients on NIV had a chance of developing OD 17.58 times more than those on conventional oxygen therapy as shown in Table 5. Patients admitted to ICU had a risk of developing OD 19.09 times more than those admitted to the ward. Multivariate analysis was done for significant factors detected by univariate analysis and illustrated that combined use of noninvasive ventilation (NIV), ICU admission, presence of OD at admission, longer duration of hospital stay, and presence of dysphonia were significant risk factors with an adjusted odds ratio (20.21, 15.28, 15.12, 1.39, and 0.150, respectively) and the overall percentage predicted OD by those factors was 88.5% (Table 5).

**Table 5:** Univariate and multivariate analyses of the risk factors of OD among the discharged patients (*N* = 310)

Variables	Total number	OD		Univariate analysis		Multivariate analysis	
				*p*	Crude odds ratio (95% CI)	*p*	Adjusted odds ratio (95% CI)
		Absent (*N* = 183)	Present (*N* = 127)				
Age (years)							
Mean ± SD	310	50.69 ± 12.50	55.81 ± 9.02	< 0.001*	1.04 (1.02–1.07)	0.140	1.04(0.99–1.10)
Gender							
Male (R)	124	77 (62.1%)	47 (37.9%)	0.371	1.24 (0.777–1.97)		
Female	186	106 (57%)	80 (43%)				
OD at admission							
Absent (R)	233	172 (73.8%)	61 (26.2%)	< 0.001*	16.92 (8.39–34.31)	0.001*	15.22(9.11–30.51)
Present	77	11 (14.3%)	66 (85.7%)				
Length of hospital stay (days)							
Median (range)	310	7 (4–20)	13 (4–42)	< 0.001*	1.28 (1.18–1.38)	< 0.001*	1.39 (1.23–1.58)
Ageusia and anosmia							
Absent (R)	253	148 (58.5%)	105 (41.5%)	0.687	0.886 (0.492–1.59)		
Present	57	35 (61.4%)	22 (38.6%)				
Dysphonia							
Absent (R)	187	154 (82.4%)	33 (17.6%)	< 0.001*	15.13 (8.63–26.51)	< 0.001*	0.15 (0.054–0.414)
Present	123	29 (23.6%)	94 (76.4%)				
NIV/IMV							
No (R)	201	163 (81.1%)	38 (18.9%)	< 0.001*	1	< 0.001*	1
NIV	102	20 (19.6%)	82 (80.4%)		17.58 (9.62–32.14)		20.21 (10.49–38.95)
IMV	7	0	7 (100%)		Undefined	0.999	Undefined
Admission							
Ward (R)	201	163 (81.1%)	38 (18.9%)	< 0.001*	19.09 (10.48–34.78)	0.002*	15.28 (9.25–33.18)
ICU	109	20 (18.3%)	89 (81.7%)				
	**Overall % predicted = 88.5%** **Model *χ***^**2**^ = 286.25, *p* < 0.001*						

*R* reference group, *NIV* Noninvasive ventilation, *IMV* Invasive mechanical ventilation

*p < 0.05: statistically significant and should be in bold

## Discussion

Oropharyngeal dysphagia is of particular concern in COVID-19 patients. It is a common problem in post-ICU patients, especially those receiving invasive mechanical ventilation, those with tracheostomies, and those with an acute chest infection, pneumonia, and respiratory insufficiency [15]. Patients with COVID-19 admitted to ward during the viral response phase, the pulmonary phase, and the hyper-inflammation phase are also at risk for oropharyngeal dysphagia [22].

The current study aimed at screening for OD in hospitalized COVID-19 patients. 500 adult patients were assessed by two valid and reliable screening tools; the Arabic EAT-10 questionnaire and Yale swallow protocol. The EAT-10, a patient-reported outcome measure (PROM) of self-perceived symptoms of OD, is recommended as an easy-to-use and quick screening tool for OD. EAT-10 was developed and validated for estimating initial OD severity and changes in response to therapy. The test is simple, takes about 2 min, and can be administered in various settings [23]. The Yale swallow protocol is an evidence-based protocol. It is the only screening tool that identifies aspiration risk and, when passed, can recommend specific oral diets without the need for further instrumental dysphagia testing. The protocol meets all the criteria necessary for a successful screening test, including being simple to administer, cross-disciplinary, cost-effective, acceptable to patients, and able to identify the target attribute by giving a positive finding when aspiration risk is present and an adverse finding when aspiration risk is absent. It was established that the sensitivity of a 3-oz challenge for the prediction of aspiration risk was 96.5%, the negative predictive value was 97.9% [24], and the false-negative rate was < 2.0% [25]. As suggested by Kimura et al. [26], in the current pandemic situation, the clinical swallowing evaluation without producing aerosols was preferred to the aerosol-generating procedures (AGPs), such as fiberoptic endoscopic evaluation of swallowing (FEES).

Our study found prevalent oropharyngeal dysphagia in hospitalized COVID-19 patients, 45.4% of admitted patients, and 40.97% of discharged patients. As Dawson et al. [27] reported, nearly 30% of COVID-19 patients admitted to the intensive care unit or the ward with respiratory problems were referred for swallowing evaluation. Dawson et al. [27] explained that dysphagia co-existed with delirium, fatigue, and difficulty accomplishing breathing/swallowing coordination. Moreover, COVID-19 has been associated with muscle weakness and increased serum creatine kinase levels [28], and this myopathy observed in skeletal muscles may affect the swallowing muscles. A higher prevalence of oropharyngeal dysphagia (51.7%) in COVID-19 patients was reported in Martin–Martinez et al. [29] study, and this could be explained by the higher mean age of their studied patients (69.3 ± 17.5 years) than our studied patients (54.2 ± 11.03 years). Oropharyngeal dysphagia is a common clinical finding in older patients with pneumonia [30], so we excluded patients over 65 years as oropharyngeal dysphagia is a highly prevalent and growing condition in the older population with the effect of aging on swallowing function. Hospitalized COVID-19 patients with a positive screen for OD were instructed to use compensatory strategies for OD management (e.g., postural changes, thickened liquids, or texture-modified food). Otherwise, the use of feeding tubes was considered.

In the present study, comparative analysis revealed that COVID-19 patients with OD showed lower oxygen saturation and higher respiratory rate than COVID-19 patients without OD. This could be due to the considerable and prolonged respiratory impairment in hospitalized COVID-19 patients, resulting in trouble coordinating breathing and swallowing. Patients with a high respiratory rate have difficulty protecting the airway during swallowing. The shortened swallowing apnea duration in patients with respiratory distress causes the larynx to open prematurely. Aguesia and anosmia were more prevalent among our COVID-19 patients with OD than patients without OD. Our results revealed that patients with ageusia and anosmia had a risk of developing OD 0.5 times more than patients with intact taste and smell function. In line with our study, Aoyagi et al. [31] reported pharyngolaryngeal sensitivity alterations, absent gag reflex, silent aspiration, and impaired pharyngeal contraction in a patient with COVID-19-related olfactory and gustatory changes and dysphagia. Our findings and Aoyagi et al. findings suggest possible impairments not only in taste and smell but also in the sensorimotor function of the pharynx and larynx, which may affect airway protection and swallowing safety.

Previous research has demonstrated that there are a variety of predictive risk factors for aspiration and dysphagia in ICU patients. Some of these risks include laryngeal damage brought on by intubation or tracheostomy, neuromuscular weakness, diminished oropharyngeal and laryngeal sensation, cognitive impairment, and poor swallowing and breathing coordination factors [32]. Similar risk factors for COVID-19 patients in the ICU have been suggested [14]. In our study, we investigated risk factors of OD in COVID-19 patients both at the time of hospital admission and discharge. We found that older age, longer duration of presenting symptoms of COVID-19, presence of ageusia and anosmia, presence of dysphonia, ICU admission, lower oxygen saturation, and higher respiratory rate were the risk factors of OD in admitted patients. For discharged patients, risk factors of OD included older age, presence of OD at admission, longer duration of hospital stay, presence of dysphonia, ICU admission, and use of NIV and/or IMV.

In the current study, ICU admission was a risk factor for OD for admitted or discharged COVID-19 patients. Such a finding is in accordance with Dawson et al. [27] study on swallowing outcomes of critically ill COVID-19 patients, where 67% of their patients evaluated in the ICU were advised to be nil by mouth. In addition, the presence of dysphonia was a risk factor for OD both for our admitted and discharged patients. This finding was supported by Osbeck Sandblom et al. [33], who observed that patients with severe COVID-19 treated in the ICU frequently had aberrant laryngeal findings and impaired oropharyngeal swallowing function. The presence of OD at hospital admission and longer duration of hospital stay were risk factors for OD for our discharged patients. These results are in harmony with Schefold et al. findings [34]. They demonstrated that almost half of critically ill patients with dysphagia continued to have dysphagia at the time of hospital discharge. Consistent with our study, Osbeck Sandblom et al. [33] mentioned that the length of time COVID-19 patients spent in the hospital overall and the number of days they spent in the ICU appeared to correlate with dysphagia severity.

Screening for OD in hospitalized COVID-19 patients is a mandatory procedure, whether for admitted or discharged patients. The combination of risk factors detected by multivariate analyses could be used as predictor models to determine patients at high risk of OD, which will need further swallowing assessment and intervention.

### Limitation

We could not differentially detect oropharyngeal dysphagia and aspiration in COVID-19 patients with instrumental swallowing assessment because of restrictions around AGPs, nor could we assess the upper airway in detail to understand the underlying breakdown of swallowing function.

## Conclusions

The present study revealed a high prevalence of oropharyngeal dysphagia (OD) among admitted (45.4%) and discharged (40.97%) COVID-19 patients. The combination of the following risk factors, "Presence of ageusia and anosmia, presence of dysphonia, longer duration of presenting symptoms of COVID-19, and higher respiratory rate" predict OD at hospital admission by an overall percentage of 93%. The combination of the following risk factors, "Presence of OD at admission, ICU admission, the use of NIV, longer duration of hospital admission, and presence of dysphonia" predict OD at hospital discharge by an overall percentage of 88.5%.

## Data Availability

Available (the data sets used and/or analysed during the current study are available from the corresponding author).

## Data Availability

The datasets generated during and/or analysed during the current study are available from the corresponding author on reasonable request.
